# Laser-driven programmable non-contact transfer printing of objects onto arbitrary receivers via an active elastomeric microstructured stamp

**DOI:** 10.1093/nsr/nwz109

**Published:** 2019-08-06

**Authors:** Hongyu Luo, Chengjun Wang, Changhong Linghu, Kaixin Yu, Chao Wang, Jizhou Song

**Affiliations:** Department of Engineering Mechanics, Soft Matter Research Center, and Key Laboratory of Soft Machines and Smart Devices of Zhejiang Province, Zhejiang University, Hangzhou 310027, China

**Keywords:** tunable adhesion, transfer printing, heterogeneous integration, laser-driven

## Abstract

Transfer printing, as an important assembly technique, has attracted much attention due to its valuable merits to develop novel forms of electronics such as stretchable inorganic electronics requiring the heterogeneous integration of inorganic materials with soft elastomers. Here, we report on a laser-driven programmable non-contact transfer printing technique via a simple yet robust design of active elastomeric microstructured stamp that features cavities filled with air and embedded under the contacting surface, a micro-patterned surface membrane that encapsulates the air cavities and a metal layer on the inner-cavity surfaces serving as the laser-absorbing layer. The micro-patterned surface membrane can be inflated dynamically to control the interfacial adhesion, which can be switched from strong state to weak state by more than three orders of magnitude by local laser heating of the air in the cavity with a temperature increase below 100°C. Theoretical and experimental studies reveal the fundamental aspects of the design and fabrication of the active elastomeric microstructured stamp and the operation of non-contact transfer printing. Demonstrations in the programmable transfer printing of micro-scale silicon platelets and micro-scale LED chips onto various challenging receivers illustrate the extraordinary capabilities for deterministic assembly that are difficult to address by existing printing schemes, thereby creating engineering opportunities in areas requiring the heterogeneous integration of diverse materials such as curvilinear electronics and MicroLED displays.

## INTRODUCTION

Transfer printing is an emerging assembly technique to transfer micro/nano-objects (i.e. inks) from one substrate (i.e. donor) to another substrate (i.e. receiver) using soft polymeric stamps [1–6]. This transfer printing technique enables the assembly of diverse materials in various structural layouts with large throughputs of thousands of objects per second and is valuable to develop advanced electronic systems such as flexible and stretchable inorganic electronics [7–11] requiring the heterogeneous integration of inorganic materials with soft elastomers, which represents one of the ongoing technology revolutions in the electronics industry. Transfer yields critically depend on the switch of the stamp/ink interfacial adhesion from strong state to weak state between picking and printing, and thus the challenge in transfer printing is to manipulate the stamp/ink interfacial adhesion in an active and robust manner.

Various approaches based on tunable dry adhesives have been utilized to develop transfer printing techniques. The kinetic approach that exploits the viscoelastic effect in the elastomeric stamp to pick up inks quickly from the donor and print them slowly onto the receiver is useful, but the low adhesion switchability (i.e. ∼3) greatly limits its broad utility [12,13]. The introduction of surface-relief microstructures on the elastomeric stamp surface can greatly enhance the adhesion switchability [14], but reliable process control in a robust manner remains an uneasy task. An interesting attempt is to use a shape memory polymer stamp instead of the elastomeric stamp to improve the reliability of the control process, which has led to a new generation of switchable adhesive with a much more easily controlled mechanism [15] and programmable capabilities [16]. Gecko-inspired adhesives with surface micro-patterns, which rely on external forces for adhesion control, are promising for transfer printing despite the expensive equipment and complex fabrication processes involved [17]. Active pneumatic elastomeric surfaces offer the capacity to transfer print devices onto various substrates at room temperature [18]. All these are contact techniques with performance critically depending on the receiver’s geometry and properties, since the printing requires contact of the stamp with the receiver.

In contrast to contact transfer printing techniques, non-contact approaches eliminate the influence of the receiver on the transfer yield and allow non-contact printing of inks onto arbitrary receivers. As the most important non-contact approaches, laser-induced forward transfer techniques have been widely used to deposit precise patterns of multi-components and have become a promising alternative to lithographic processes [19]. In these approaches, a pulse laser induces a strong light-matter interaction locally at the interface of film-coated substrate with or without an absorbing layer close to the receiver. Above the incident-laser-energy threshold, a small pixel of thin film is ejected from the substrate to the receiver. Translation of the receiver or scanning of the laser beam enables complex pattern formation without the necessity for a shadow mask, even under standard laboratory conditions. Despite these merits, laser-induced forward transfer relies on the evaporation or melting of film or the absorbing layer, which may induce an undesired high temperature increase (a few hundred degrees centigrade) in the system. Thanks to the rapid and localized heat delivery by the laser, this high temperature has limited the heat effect on electronic components [20]. Moreover, the interfacial damage is permanent, indicating that the laser-induced forward transfer process is not reversible, which limits its broad utility in the transfer printing of brittle materials (e.g. Si) widely involved in conventional electronics. Ideal non-contact transfer printing should offer easily controlled tunable adhesion with a high reversibility and a low temperature increase. A good attempt is to control the interfacial adhesion via interfacial thermal mismatch due to the laser heating of inks to facilitate the delamination of inks from the stamp [21–23]. Although the stamp features the simplest form of a block, this passive design has an intrinsic limitation of requiring a high temperature of 200–300°C to reach the sufficient thermal mismatch for the ink release. This unfavorably high temperature increase may damage the stamp/ink interface and thus reduce the reliability of the process significantly. The laser-driven shape memory effect has been explored to reduce the temperature increase for transfer printing heat-sensitive material and reflective material [20].

Here, we report a laser-driven programmable non-contact transfer printing technique via the simple yet robust design of an active elastomeric microstructured stamp with tunable adhesion. The tunable adhesive features cavities filled with air and encapsulated by a micro-patterned surface membrane duplicated from low-cost and easily available sandpapers. The micro-patterned surface membrane can be inflated dynamically to control the interfacial adhesion by heating the air in cavities through a metal layer (e.g. iron particles) on the inner cavity surface, which serves as the laser absorbing layer. This construct offers continuously thermal-controlled tunable adhesion with a large switchability of more than three orders of magnitude at a temperature increase below 100°C. This active adhesive is completely different from those previously reported, which are impossible for developing reliable programmable non-contact transfer printing. Theoretical and experimental studies reveal the fundamental aspects of the design and fabrication of the active elastomeric microstructured stamp, and the operation of non-contact transfer printing. Demonstrations in transfer printing of micro-scale Si platelets and micro-scale LED chips onto various challenging receivers illustrate the unusual capabilities for deterministic assembly.

## RESULTS AND DISCUSSION

### Active elastomeric stamp design and laser-driven programmable non-contact transfer printing

**Figure 1.**
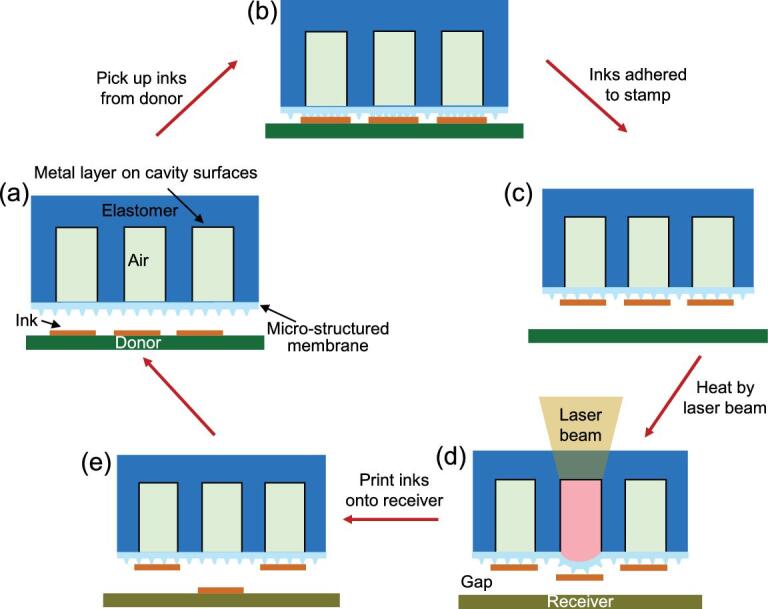
Schematic illustration of the laser-driven programmable non-contact transfer printing process via an active elastomeric microstructured stamp. (a) The stamp is moved above the donor. (b) The stamp is in contact with the inks by a light pressure with the surface micro-tips collapsed. (c) The rapid retraction retrieves the inks onto the stamp with the surface micro-tips recovered. (d) The inked stamp is moved on top of the receiver, leaving a small gap between the stamp and the receiver. A programmable laser beam is used to heat the air in the stamp cavities, which induces pressures on the microstructured stamp membranes to facilitate the delamination of inks from the stamp. (e) The removal of the stamp completes the transfer printing process. Inks on the unheated region remain on the stamp while those on the heated region are printed onto the receiver.

Figure 1 schematically illustrates the typical process of the laser-driven programmable non-contact transfer printing via the active elastomeric microstructured stamp. The stamp has circular cavities filled with air and encapsulated by micro-patterned surface membranes (Fig. 1a), which can be inflated dynamically to control the interfacial adhesion by heating the air in cavities through the laser-absorbing metal layer on the inner-cavity surfaces. The transfer printing process consists of two steps: retrieval and programmable printing. For the retrieval, the stamp is moved above the donor (Fig. 1a) followed by intimate contact with the inks to be transferred. Without heating, the stamp membrane remains flat with the micro-tips collapsed, enabling conformal contact between the stamp and the inks, yielding a relatively strong adhesion (Fig. 1b). Rapid retraction of the stamp maximizes the adhesion due to the viscoelastic effect and ensures the retrieval of inks from the donor (Fig. 1c). For the programmable printing, the inked stamp is moved above the receiver by leaving a small gap between the stamp surface and the receiver, which allows the non-contact printing. After the laser alignment, the air in the cavities is heated quickly for inflation to induce a pressure on the stamp membrane and form a bulge, which ensures weak adhesion and facilitates the ejection of inks from the stamp (Fig. 1d) to the receiver. Translation of the receiver enables the programmable printing of inks on the heated region onto the receiver, while inks on the unheated region remain on the stamp. The retraction of the stamp completes the transfer printing process (Fig. 1e), with the bulged membrane recovering back to a flat one.

Here, the active elastomeric microstructured stamp incorporates three critical components: (i) the cavities filled with air and embedded under the contacting surface, (ii) the micro-patterned membrane that encapsulates the air cavities and serves as the tunable adhesive and (iii) the metal layer on the inner-cavity surfaces that serves as the laser-absorbing layer. These three components yield the laser-driven programmable adhesive, which is critical to develop laser-driven programmable non-contact transfer printing. The bulk of the stamp with the embedded cavities and the micro-patterned membrane can be fabricated by the twice replica method (Supplementary Fig. 1a and b) from polydimethylsiloxane (PDMS, Dow Corning Sylgard 184) with 10:1 monomer:cross-linking agent. The low-cost and easily available sandpaper is taken as the mold to fabricate the micro-patterns on the membrane, which bypasses the complex lithographic processes. Iron particles are utilized to cover the inner-cavity surfaces as the laser-absorbing layer, which avoids the use of expensive micro-fabrication equipment to deposit the thin metal layer. The stamp membrane is then bonded to the stamp with cavities after plasma treatment (Supplementary Fig. 1c). These processes establish cost-effective and practical routes to fabricate the active elastomeric microstructured stamp.

### Adhesion characteristics of the active elastomeric microstructured stamp

**Figure 2.**
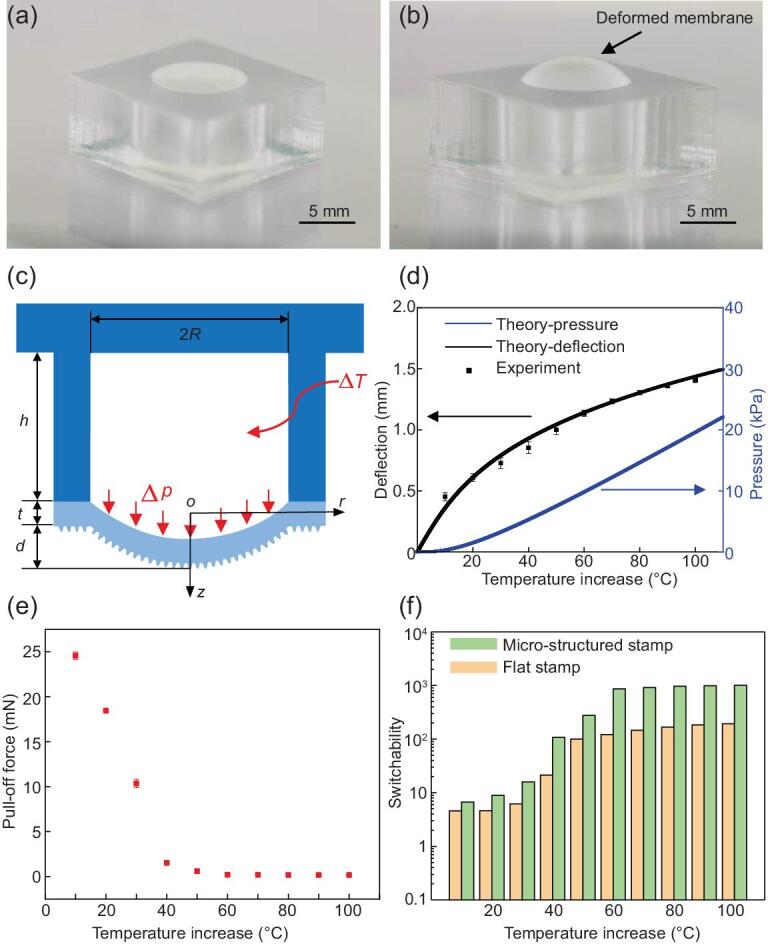
The active elastomeric microstructured stamp with a macro-scale cavity and its adhesion characteristics. (a) Photograph of the unheated microstructured stamp with the stamp membrane remaining flat (stamp height: 6 mm; stamp width: 16 mm; cavity diameter: 7 mm; membrane thickness: 118 μm). (b) Photograph of the heated microstructured stamp with the stamp membrane deformed to bulge. (c) Illustration of the cross-section of the deformed microstructured stamp when heated by the laser beam. (d) The membrane deflection and cavity pressure as the functions of the temperature increase. (e) The pull-off force as the function of the temperature increase. (f) The adhesion switchability versus the temperature increase for the microstructured stamp and flat stamp.

Figure 2 shows the active elastomeric microstructured stamp with a macro-scale cavity and its adhesion characteristics. Fig. 2a provides a photograph of a representative stamp of this type, which incorporates a circular cavity (7 mm in diameter and 6 mm in depth) in a square elastomeric PDMS stamp (16 × 16 × 7 mm) filled with air and encapsulated by a micro-patterned 118-μm-thick PDMS membrane obtained by duplicating a cheap sandpaper (2000 mesh), which yields irregular micro-patterns with surface heights to the order of 10 μm. The stamp is finally bonded to a glass backing (70 × 30 × 1 mm) for easy handling. To reveal the underlying physics associated with the adhesion control, the air in the cavity is heated by a thermal stage, thus the laser-absorbing layer is not included in this stamp. When the stamp is heated, the air in the cavity inflates to increase the cavity pressure, which bulges the stamp membrane (Fig. 2b). We denote the pressure increase in the cavity as the inflation pressure.

To quantify the relations of the inflation pressure in the cavity and the membrane deflection with the temperature increase, a theoretical mechanics model is established. Fig. 2c illustrates the cross-section of the active stamp when heated, with key parameters and dimensions labeled. The circular cavity has a radius of *R* and a depth of *h*. When heated, the membrane, whose Young modulus is *E* and thickness is *t*, has a maximum deflection of *d*. The temperature increase of the air in the cavity is Δ*T*, which causes an inflation pressure of Δ*P*. A cylindrical coordinate system with the coordinates (*r*, *z*) and the origin *O* located at the center of membrane is established as shown in Fig. 2c. The stamp membrane can be modeled as a thin clamped circular plate under the uniform inflation pressure Δ*P* with the membrane deflection *w*(*r*) given by [24]:


(1) \begin{eqnarray*} && \, w(r)&=&{\left(\frac{R^4\Delta P}{Et}\right)}^{\frac{1}{3}}{\left(\frac{c}{2}\right)}^{\frac{1}{3}}\nonumber\\ &&\times\left[g(c)-g\left(\frac{cr^2}{R^2}\right)\frac{r^2}{R^2}\right], \end{eqnarray*}

where $g(x)=1+\frac{1}{4}x+\frac{5}{36}{x}^2$ and *c* is a dimensionless parameter depending on the Poisson’s ratio. The maximum membrane deflection is given by:


(2) \begin{equation*} d={\left(\frac{cR^4\Delta P}{2 Et}\right)}^{\frac{1}{3}}g(c). \end{equation*}

The volume change of the cavity can then be obtained by:


(3) \begin{eqnarray*}\Delta V &=& {\int}_R^0\pi {r}^2{w}^{\prime }(r) dr\nonumber\\ &=&\ \pi {R}^2{\left(\frac{R^4\Delta P}{Et}\right)}^{\frac{1}{3}}{\left(\frac{c}{2}\right)}^{\frac{1}{3}}\left(\frac{1}{2}+\frac{c}{6}+\frac{5{c}^2}{48}\right).\nonumber\\ \end{eqnarray*}

The equation of ideal gas gives:


(4) \begin{eqnarray*} \frac{P_0{V}_0}{T_0}=\frac{\left({P}_0+\Delta P\right)\left({V}_0+\Delta V\right)}{T_0+\Delta T}, \end{eqnarray*}

where ${V}_0=\pi {R}^2h$ is the initial volume of the cavity, *T*_0_ is the initial temperature of the air in the cavity and *P*_0_ is the initial atmospheric pressure. Substituting Equation 3 into Equation 4 yields the relation between Δ*P* and Δ*T* as:


(5) \begin{eqnarray*} 1+\frac{\Delta T}{T_0}&=&\left(1+\frac{\Delta P}{P_0}\right)\Bigg[1+\frac{R}{h}{\left(\frac{R\Delta P}{Et}\right)}^{\frac{1}{3}}\nonumber\\ &&\times{\left(\frac{c}{2}\right)}^{\frac{1}{3}}\left(\frac{1}{2}+\frac{c}{6}+\frac{5{c}^2}{48}\right)\Bigg]. \end{eqnarray*}

Then, Equations (2) and (5) give the relation between the maximum membrane deflection *d* and Δ*T*.

Figure 2d shows the dependences of the maximum membrane deflection and the inflation pressure on the temperature increase under ${P}_0=101.3\ \mathrm{kPa}$ and ${T}_0=293.15\ \mathrm{K}$. For the PDMS stamp with the Poisson’s ratio of 0.49, *c* is 0.318. The PDMS modulus is measured as 1.7 MPa. The good agreement between the theoretical predictions (black solid line) and experimental measurements (black solid dot) on the maximum membrane deflection validates the accuracy of the theoretical model. More importantly, as the temperature increases, both the inflation pressure in the cavity and the membrane deflection increase significantly. For example, as the temperature increases from 20°C to 120°C, the inflation pressure in the cavity can reach 19.58 kPa (∼20% atmospheric pressure), which is large enough to induce a noticeable deflection of 1.41 mm (∼20% of the cavity diameter). The high inflation pressure and the large deflection ensure a reliable way to tune the interfacial adhesion under a low temperature increase (∼100°C).

To measure the adhesion of the active elastomeric stamp, vertical pull tests were carried out under various temperature increases for a stamp/glass interface. The measurement set-up (Supplementary Fig. 2) consists of a Materials Testing System (Model 5944, INSTRON), a two-axis manual tip/tilt platform and a thermal stage. The glass substrate is fixed to the two-axis manual tip/tilt platform, which is connected to the load cell. The active elastomeric stamp is placed onto the thermal stage. During the pull test, the glass substrate approaches and contacts with the stamp at a fixed speed of 10 μm/s. To demonstrate the adhesion tunability of the active elastomeric stamp, an appropriate small preload of 150 mN is selected in experiments. After a relaxation time of 10 s, the glass is retracted at a controlled velocity of 500 μm/s. The maximum force from the resulting force–displacement curve recorded by the load cell gives the pull-off force. It should be noted that the adhesion does not exist if the non-contact printing occurs.

Figure 2e shows the measured pull-off force under various temperature increases. As expected, the pull-off force decreases with the temperature increase. The weakening of the interfacial adhesion at the high temperature is due to the bulge of the stamp membrane, which reduces the contact area and thus the interfacial adhesion. Below 40°C, the pull-off force decreases almost linearly and dramatically, while, above 40°C, the pull-off force decreases slowly due to the negligible difference in the change of contact area at a high temperature increase. Fig. 2f shows the adhesion switchability, defined as the maximum adhesion strength over the minimum adhesion strength (or the maximum pull-off force over the minimum pull-off force), as the function of the temperature increase for the active stamp and flat stamp. The adhesion switchability increases with the temperature increase, since the maximum pull-off force occurs at zero temperature increase and remains unchanged, while the minimum pull-off force reduces with the temperature increase. Compared to the flat stamp, whose adhesion switchability is up to two orders of magnitude, the adhesion switchability of the active stamp can reach as high as three orders of magnitude with a temperature increase of below 100°C, which results from the micro-patterns on the stamp membrane reducing the interfacial adhesion dramatically. This powerful thermal-controlled adhesion-modulation capability of the active elastomeric microstructured stamp is of great importance, making non-contact printing possible.

### Transfer printing of silicon platelets onto various challenging receivers

**Figure 3.**
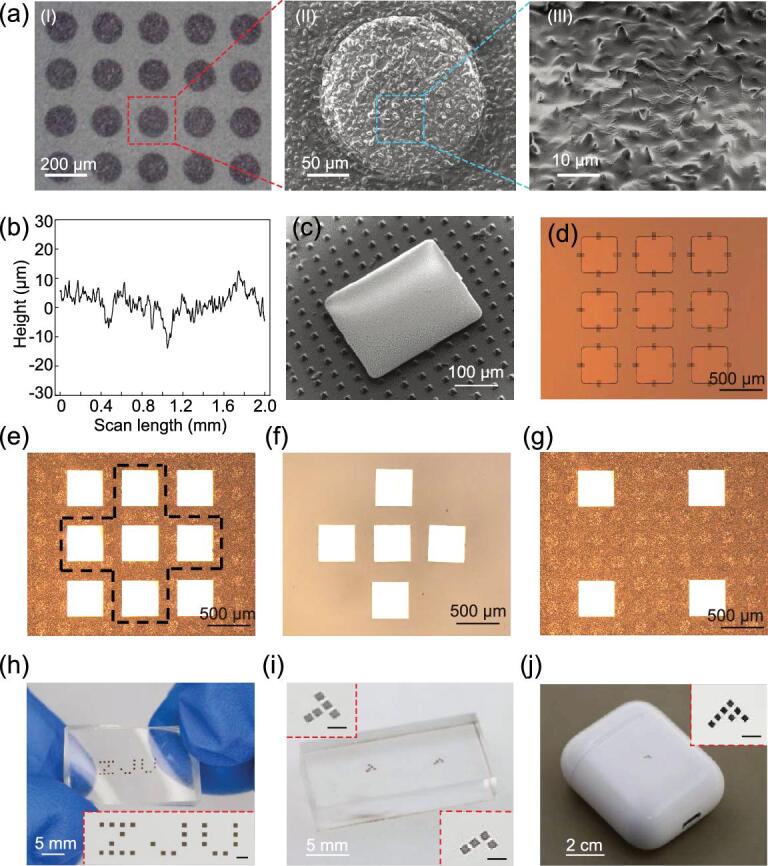
Demonstrations of the microstructured stamp with micro-scale cavities in programmable printing Si platelets (350 × 350 × 3 μm) onto various receivers. (a) Microscopy images of the microstructured stamp surface (stamp height: 2 mm; stamp width: 16 mm; cavity diameter: 160 μm; membrane thickness: 25 μm). (I) Optical, (II) scanning electron microscope (SEM) and (III) SEM images of the micro-structure stamp surface. (b) Surface morphology of the microstructured stamp obtained by a Stylus Profiler. (c) SEM image of a Si platelet printed onto PDMS with pyramid microstructures. (d) 3 × 3 Si platelets on SOI. (e) 3 × 3 Si platelets on the microstructured stamp after the pick-up with those surrounded by the dotted line to be heated by the laser beam. (f) The printed cross pattern on PDMS. (g) The remaining Si platelets on the stamp. (h) The printed ‘ZJU’ pattern on PDMS. (i) Si platelets printed onto acrylic to form a letter ‘T’ and a letter ‘L’. (j) Si plates printed onto the cover of Apple AirPods to form a letter ‘F’. Scale bars of insets of (h), (i) and (j) correspond to 1 mm.

In order to demonstrate the capability of the active elastomeric microstructured stamp in the transfer printing of micro-scale inks onto arbitrary receivers, an active elastomeric stamp with micro-scale circular cavities was fabricated. Fig. 3a shows the microscopy images of the active stamp surface. The cavity has the dimensions of 160 μm in diameter and 350 μm in depth. The spacing between the centers of neighboring cavities is 240 μm. The stamp membrane (25 μm thick) is duplicated from a cheap sandpaper (2000 mesh), which yields irregular micro-patterns with surface heights in the order of 10 μm, as shown in the typical surface morphology of a stamp membrane (Fig. 3b). This method of fabricating micro-patterns greatly reduces the cost while achieving the desired function without involving any micro-fabrication processes (e.g. photolithography and etching), which were required in previous work [25–27] to fabricate the well-organized micro-patterns. A laser beam (∼808 nm wavelength and 400 μm spot diameter) with a low power of 400 mW from a laser-generation system (FC-W-808 nm-10 W, Changchun New Industries Optoelectronics Tech. Co., Ltd) is sufficient to enable non-contact printing. The delamination of ink could occur within 100 ms (see Supplementary Movie 1), which is essential to achieve a high assemble rate.

Micro-scale silicon platelets (350 × 350 × 3 μm) with fabrication details given in the Experimental Section are taken as inks to demonstrate the non-contact-printing capability of the active elastomeric microstructured stamp. Fig. 3c shows a silicon platelet printed onto PDMS with pyramid micro-tips, which demonstrates the unusual ability of printing micro-scale inks onto rough and uneven surfaces with ultra-low adhesion. With the aid of local laser heating, programmable printing in any desired pattern can be realized. As a simple demonstration, a cross pattern is printed onto PDMS, with the key results presented in Fig. 3d–g. Fig. 3d shows the optical microscopy image of a 3 × 3 array of silicon platelets on silicon-on-insulator (SOI) before transfer printing. The array of the silicon platelet is retrieved by the active elastomeric stamp with those surrounded by the dotted line to be printed onto PDMS (Fig. 3e). The printed silicon platelets due to the local laser heating forms a cross pattern on PDMS (Fig. 3f) while the silicon platelets without laser heating still remain on the stamp (Fig. 3g). Some misalignments (Fig. 3e) can be observed due to the large gap between the inks and receivers, the non-uniform distribution of the micro-patterns and the non-uniform laser-power distribution over the laser spot. Fig. 3h shows the optical image of a ‘ZJU’ pattern on PDMS. Fig. 3f and 3g shows silicon platelets printed onto acrylic to form the letters ‘T’ and ‘L’ and the cover of Apple AirPods to form a letter ‘F’, respectively, which demonstrate the unusual ability of programmable printing micro-scale inks onto smooth surfaces with ultra-low adhesion. The above examples demonstrate the extraordinary capabilities of the programmable non-contact transfer printing of fragile inks onto arbitrary surfaces, which is beyond the capabilities of existing contact approaches [4,12].

### Transfer printing of micro-scale LED chips onto various challenging receivers

**Figure 4.**
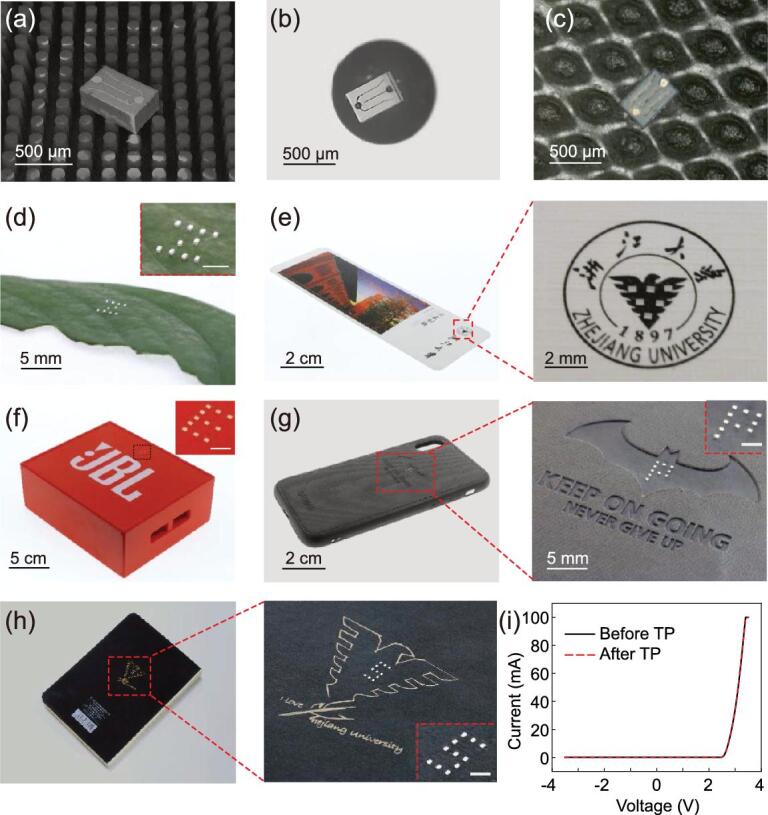
Demonstrations of printing micro-scale LED chips (400 × 200 × 90 μm) onto unusual and challenging receivers. Printing a single LED chip onto (a) PDMS pillar arrays and (b) a steel sphere with a diameter of 1 mm and a piece of paper (c). Programmable printing LED chips onto (d) a leaf to form a letter ‘Z’, (e) a postcard with a triangle pattern, (f) a Bluetooth speaker to form a letter ‘E’, (g) a mobile-phone shell to form a letter ‘P’ and (h) a notebook to form a letter ‘S’. (i) Electrical properties of LED chips before and after transfer printing onto the notebook. Scale bars of insets of (d), (f), (g) and (h) correspond to 1 mm.

Examples of enhanced capabilities of printing functional electronic components enabled by the active elastomeric microstructured stamp are shown in Fig. 4, in which micro-scale LED chips (400 × 200 × 90 μm) are printed onto a variety of unusual surfaces not easily accessible through other schemes. Fig. 4a–c shows the capability of printing a single micro-scale LED chip onto various adhesiveless planar or curvilinear surfaces, such as a PDMS pillar array with a pillar diameter of 100 μm and a pillar spacing of 150 μm (Fig. 4a), a steel sphere with a diameter of 1 mm (Fig. 4b) and a piece of paper (Fig. 4c).

Programmable printing of micro-scale LED chips onto various challenging receivers is demonstrated in Fig. 4d–h. Fig. 4d shows LED chips printed onto the uneven and curvilinear surface of a scindapsus leaf to form a letter ‘Z’, which is potentially relevant to the integration of functional devices with living systems. Fig. 4e shows micro-scale LED chips printed onto a commercially available postcard to form an inverted-triangle pattern, which could be of value for paper-based electronics. Fig. 4f provides a similar demonstration of micro-scale LED chips printed onto a Bluetooth speaker to form a letter ‘E’. The surfaces of the postcard and Bluetooth speaker are smooth with ultra-low adhesion, which are difficult to print onto using existing contact-printing schemes. Fig. 4g shows micro-scale LED chips printed onto a mobile-phone shell with a pit to form a letter ‘P’, which is not possible through other transfer printing schemes. Fig. 4h shows micro-scale LED chips printed onto a notebook with the Zhejiang University logo to form a letter ‘S’. Fig. 4i compares the performance of the micro-scale LED chip before and after transfer printing it onto the notebook. The consistency of the voltage-current curves before and after printing indicates that our laser-driven programmable transfer printing technique does not influence the performance of micro-scale LED chips at all. The programmable non-contact-printing capabilities permit the printing of micro-scale LED chips in any desired pattern onto any receivers, which is valuable for the assembly of micro-scale LED chips for MicroLED display.

It should be noted that the theoretical mechanics model in this work was established based on a quasi-static hypothesis. The dynamic effect of laser heating is helpful to promote the printing process, since it induces a larger membrane deflection, which further weakens the interfacial adhesion. For the case of a micro-scale cavity with a depth *h* of 350 μm in the experiments, the thermal response time of the air (thermal diffusivity α: 2 × 10^−5^ m^2^/s) in the cavity determined by *h*^2^/α is in the order of 10 ms. Based on finite element simulations, the membrane deflection can increase by 10% for full loading within 10 ms. This increase in the membrane deflection is helpful to promote the printing process. Although our approach is good for Si platelets and inorganic LED chips with thicknesses larger than 1 μm, it is still challenging for the transfer printing of ultra-thin materials (e.g. graphene) with nanoscale thicknesses or weak binding energy since they may deform with the stamp membrane due to the ultra-low stiffness. Some researchers have adopted thermal and/or local force control for the transfer printing of ultra-thin nanomaterials such as graphene and MoS_2_ [28] or an ultra-thin nanoparticle assembly layer [29].

## CONCLUSION

Here, we have reported on a laser-driven programmable non-contact transfer printing technique via a simple yet robust design of an active elastomeric microstructured stamp with tunable adhesion. The tunable adhesive, which offers an adhesion switchability of more than three orders of magnitude with a temperature increase of below 100°C, features cavities filled with air and embedded under the contacting surface, a micro-patterned membrane that encapsulates the air cavities and serves as a tunable surface and a metal layer on the inner cavity surfaces that serves as the laser-absorbing layer. The fabrication of the adhesive bypasses the complex lithographic processes and provides a cost-effective route to realize the active elastomeric microstructured stamp. Demonstrations in the programmable transfer printing of micro-scale Si platelets and micro-scale LED chips onto various challenging flat or rough receivers (e.g. paper, steel sphere, leaf) with ultra-low adhesion illustrate the unusual capabilities for deterministic assembly that have been difficult to address by existing printing schemes, although the transfer printing of materials with nanoscale thickness or weak binding energy is still challenging. This innovative laser-driven non-contact transfer printing technique creates engineering opportunities in a wide range of applications such as flexible electronics, paper-based electronics, bio-integrated electronics and MicroLED displays, where the heterogeneous integration of diverse materials is required.

## METHODS

### Preparation of the stamp cavities

The fabrication process is schematically illustrated in Supplementary Fig. 1a. Initially, a liquid PDMS mixture (10:1 monomer:cross-linking agent) was poured against a photolithographically patterned Si template. The PDMS was degassed in a vacuum chamber for 30 min and then cured in an oven at 75°C for 4 h, cooled and demolded from the template. After demolding, a PDMS positive mold was generated and exposed to a UV/ozone environment for 30 min to form a non-stick oxide layer on the surface. Then, the liquid PDMS mixture (10:1 monomer:cross-linking agent) was poured into the positive PDMS mold, degassed in a vacuum chamber for 30 min and then cured in an oven at 75°C for 4 h. Finally, the stamp cavities were demolded from the positive PDMS mold.

### Preparation of the micro-patterned stamp membrane

At the beginning, a liquid PDMS mixture (10:1 monomer:cross-linking agent) was poured onto a sandpaper (2000-mesh) template. The PDMS was degassed in a vacuum chamber for 30 min and then cured in an oven at 75°C for 4 h, cooled and demolded from the template. After demolding, a PDMS positive mold was generated and exposed to a UV/ozone environment for 30 min to form a non-stick oxide layer on the surface. The thin microstructured membrane was formed (Supplementary Fig. 1b) by spin coating (3000 RPM, 30 s) a PDMS mixture (10:1 monomer:cross-linking agent) onto the PDMS positive mold and cured in an oven at 75°C for 4 h.

### Assembly of the active elastomeric stamp

Initially, the stamp cavities were filled with pure iron particles (10 nm in diameter). Then, an air duster was used to remove the iron particles, leaving those adhered on the surface of the cavities to serve as the laser-absorbing layer. The bottom surfaces of the stamp reservoir and the stamp membrane were activated using plasma etching techniques (Supplementary Fig. 1c). The two activated surfaces were then brought into contact and the active elastomeric stamp was peeled off the PDMS positive mold with the membrane chemically bonded to the stamp body.

### Adhesion measurements

The interfacial adhesion of the stamp against a flat glass substrate was measured by pull tests using a home-made measurement set-up (Supplementary Fig. 2) consisting of a Materials Testing System (Model 5944, INSTRON), a manual tip/tilt platform and a thermal stage. The manual tip/tilt platform facilitates alignment between the stamp and the glass substrate. The temperature increase is controlled by the thermal stage. The glass substrate is brought into contact with the glass substrate with a 150 mN preload and then retracted at a speed of 500 μm/s.

### Fabrication of silicon platelets

Supplementary Fig. 3 shows the procedure to fabricate silicon platelets. These platelets were derived from SOI wafers with 3 μm thick device layers (top silicon). A layer of the photoresist pattern is prepared on the surface of the SOI, which serves as a resist for reactive ion etching of the top silicon. After inductive coupled plasma (ICP) etching, the silicon platelets in printable configurations are fixed using a photoresist anchor. Bathing the chip in concentrated hydrofluoric acid removed the oxide layer. The silicon platelets were picked up by the stamp for printing.

### Automated transfer printing

The automated transfer printing platform is shown in Supplementary Fig. 4. The platform incorporates an electric displacement console, a microscopic imaging system and a laser-generation system. The stamp and the receiver substrates are placed on the displacement console with a small gap (50–150 μm) between them. Then, the laser beam is focused on the target area using the microscopic imaging system. Programmable non-contact transfer printing is realized by controlling the displacement console with a computer program.

## Supplementary Material

nwz109_Supplemental_Files
