# Supplementary material for: Laser-driven programmable non-contact transfer printing of objects onto arbitrary receivers via an active elastomeric microstructured stamp
Source: Natl Sci Rev. 2019 Aug 6;7(2):296–304. doi: 10.1093/nsr/nwz109 (PMC8288994; doi:10.1093/nsr/nwz109)
Supplement: nwz109_Supplemental_Files [file nwz109_supplemental_files.zip › SI.docx]

Supporting Information

**Laser-Driven Programmable Non-Contact Transfer Printing of Objects onto Arbitrary Receivers via an Active Elastomeric Micro-Structured Stamp**

Hongyu Luo, Chengjun Wang, Changhong Linghu, Kaixin Yu, Chao Wang, Jizhou Song^*^

Department of Engineering Mechanics, Soft Matter Research Center, and Key Laboratory of Soft Machines and Smart Devices of Zhejiang Province, Zhejiang University, Hangzhou 310027, China

^*^ Corresponding author. Email: jzsong@zju.edu.cn

**Figure S1**


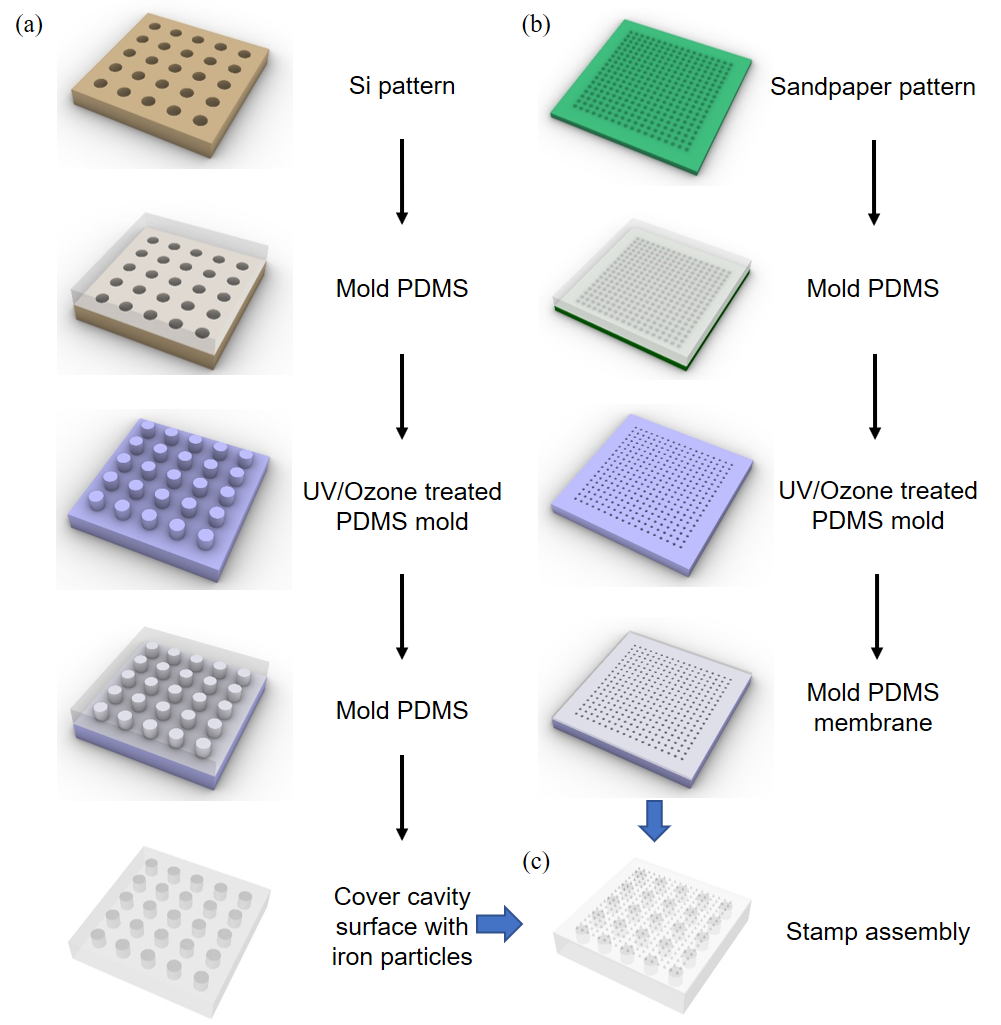


**Figure S1.** Schematic illustration of the fabrication of the active elastomeric stamp. (a) The fabrication process of cavities embedded in PDMS stamp body using a twice replica method. The inner cavity surfaces are covered by iron particles. (b) The fabrication process of stamp membrane using a twice replica method. (c) The bond of stamp membrane to the stamp body by plasma treatment to obtain the active elastomeric stamp.

**Figure S2**


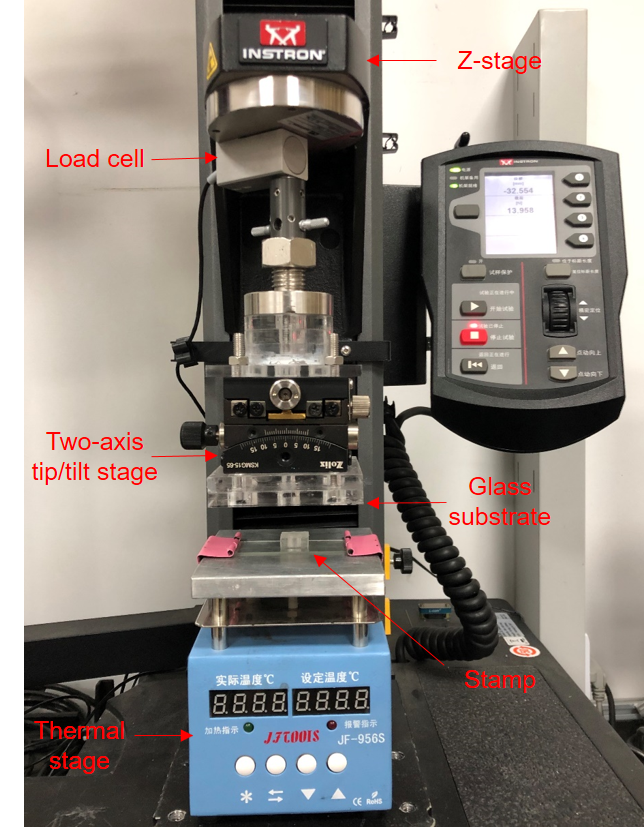


**Figure S2.** Photograph of the home-made pull test equipment, which consists of a Materials Testing System (Model 5944, INSTRON), a manual tip/tilt platform, and a thermal stage.

**Figure S3**


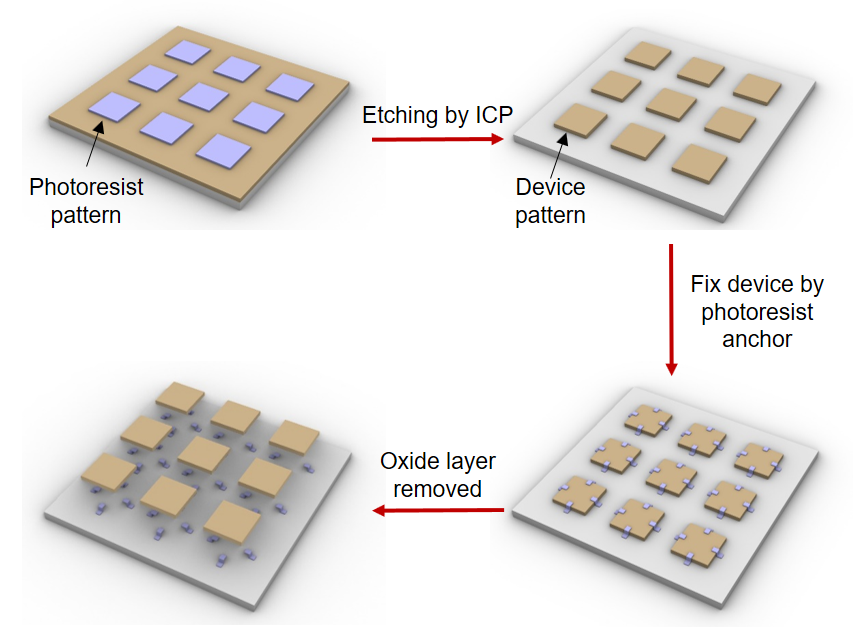


**Figure S3.** Schematic illustration of the process for fabricating silicon platelets in printable configurations, starting with silicon-on-insulator (SOI) wafers with 3 µm thick top Si layer.

**Figure S4**


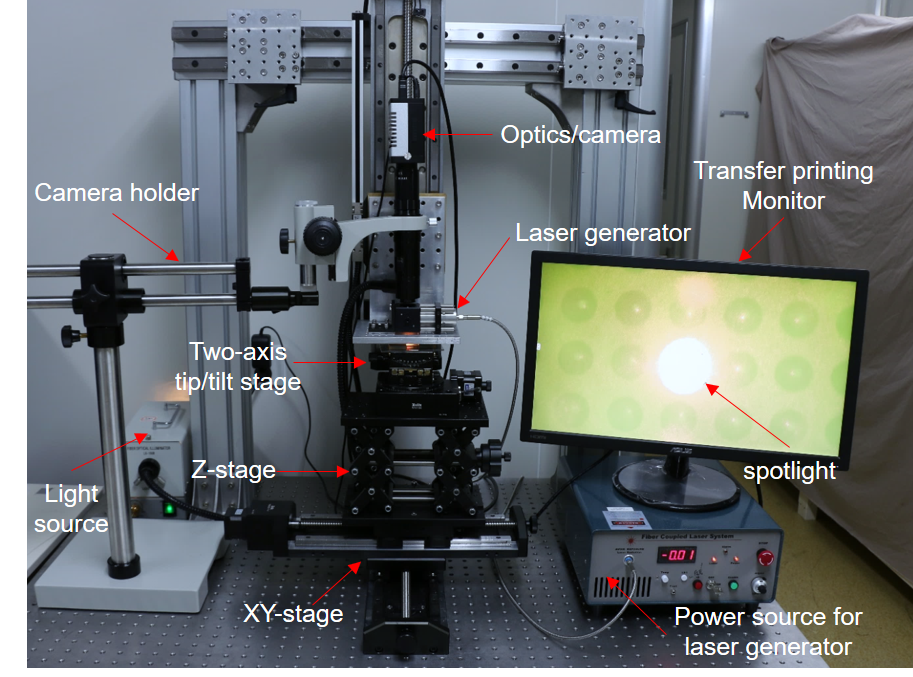


**Figure S4.** Photography of the automated laser-driven transfer printing platform, which incorporates an electric displacement console, a microscopic imaging system and a laser generation system.
